# Comparative Phenotypical and Molecular Analyses of Arabidopsis Grown under Fluorescent and LED Light

**DOI:** 10.3390/plants6020024

**Published:** 2017-06-13

**Authors:** Franka Seiler, Jürgen Soll, Bettina Bölter

**Affiliations:** Department Biologie I-Botanik, Ludwig-Maximilians-Universität, Großhadernerstr. 2-4, Planegg-Martinsried 82152, Germany; f.andersch@lmu.de (F.S.); soll@lmu.de (J.S.)

**Keywords:** Arabidopsis, light quality, LED, phenotype, gene expression

## Abstract

Comparative analyses of phenotypic and molecular traits of *Arabidopsis thaliana* grown under standardised conditions is still a challenge using climatic devices supplied with common light sources. These are in most cases fluorescent lights, which have several disadvantages such as heat production at higher light intensities, an invariable spectral output, and relatively rapid “ageing”. This results in non-desired variations of growth conditions and lowers the comparability of data acquired over extended time periods. In this study, we investigated the growth behaviour of Arabidopsis Col0 under different light conditions, applying fluorescent compared to LED lamps, and we conducted physiological as well as gene expression analyses. By changing the spectral composition and/or light intensity of LEDs we can clearly influence the growth behaviour of Arabidopsis and thereby study phenotypic attributes under very specific light conditions that are stable and reproducible, which is not necessarily given for fluorescent lamps. By using LED lights, we can also roughly mimic the sun light emission spectrum, enabling us to study plant growth in a more natural-like light set-up. We observed distinct growth behaviour under the different light regimes which was reflected by physiological properties of the plants. In conclusion, LEDs provide variable emission spectra for studying plant growth under defined, stable light conditions.

## 1. Introduction

Light is the most important parameter for plant growth and development. Not only the intensity of light, but also the spectral quality has a great effect on many aspects of plant life such as photosynthetic performance, differentiation, and flowering [1]. Therefore, plants feature several sensors for light quantity, day length (photoperiod), and spectral quality [2]. These sensors (photoreceptors) are at the root of complex signalling networks, which control developmental, physiological, and morphological processes. The range of wavelengths that can be utilized by plants for different purposes ranges from 280 nm to 750 nm [3]. Within this spectrum, 380–730 nm covers the visible light, which is absorbed mainly by chlorophyll a, b, and carotenoids. Violet, blue, and red light play a great role in photosynthesis, whereas red and far-red light (730 nm) also influence germination, vegetative growth, budding, and flowering [4]. Several classes of photoreceptors have been described: phytochromes (in Arabidopsis there are five family members called phyA-phyE) absorb red and far-red light, whereas blue light is perceived by cryptochromes (cry.1 and cry.2), phototropins (phot.1 and phot.2), and Zeitlupes (ZKL, FKF1 and LKP2) [2]. UV-B radiation can be sensed via specific receptors called UVR8. Downstream signalling first and foremost leads to stress-related adaptations which results in the protective measures against the harmful UV-B radiation [5]. Virtually all processes within a life cycle of a plant are initiated and/or regulated by light via perception by photoreceptors and their downstream signalling cascades. This often involves the (de-) activation of transcription factors, which influence the expression of genes e.g., associated with hormone synthesis/transport. 

One prominent example is the red and far red light-absorbing phytochrome family that is critically involved in photomorphogenesis, the developmental response of an organism to the information contained in light. This process includes slowed stem elongation, cotyledon/leaf expansion, and greening and straightening of the apical hook. Characteristically, phytochromes occur in an inactive (Pr) and active (Pfr) form, which are interconvertible via conformational changes upon absorption of red or far red light, respectively. Red light (R) induces the active conformation (Pfr), which is translocated to the nucleus, where phytochromes inhibit two different classes of repressive transcription factors. PIFs (phytochrome interacting factors) directly bind to Pfr forms of phytochromes upon which these are phosphorylated followed by ubiquitination and degradation by the proteasomal system. The removal of these and other repressive transcription factors allows the initiation of photomorphogenesis. The molecular details of the complex signalling cascades include massive differential gene regulation and are still not fully understood [6]. Further processes influenced by red/far red light are the shade avoidance response and flowering initiation [2]. Induction of shade avoidance is mediated by a lowered R:FR light ratio, which in fact represents reflected far red light by neighbouring vegetation. Since the red light amount remains constant, the ratio drops and that signals probable imminent shading to the receiving plant which then initiates the avoidance response [7].

Blue light acts via cryptochromes, which also control many developmental processes in plants, specifically de-etiolation, elongation, flowering, and maintenance of the circadian clock. Cryptochromes also play an important role in photomorphogenesis. They derive from ancient DNA repair enzymes known as photolyases. Though cryptochromes no longer fulfil DNA repair-related functions, they are structurally still very similar to their progenitors and have kept FAD as a co-factor. Illumination with blue light seems to induce continuous cycling between different redox states of FAD, which correlates with biological activity. In Arabidopsis, both cryptochrome isoforms (Cry.1 and Cry.2) were localised to the nucleus, but Cry.1 was also found in the cytosol where it seemed to be involved in different regulatory processes than the nuclear localised form [8]. 

Natural sun light covers the complete range of usable wavelengths, representing an ideal light source, whereas the usually applied illuminants in plant breeding and research are fluorescent, metal-halide, high-pressure sodium, or incandescent lamps. These not only contain wavelengths dispensable for the plant, mainly in the green light area, but also consume huge amounts of energy and generate a great measure of unwanted heat. In comparison, light-emitting diodes (LEDs) have several advantageous features, such as the possibility to control spectral composition and therefore mimic the natural light as closely as possible, a long lifespan, small mass and volume, and negligible heat emission combined with low energy consumption [9]. Though the first reports about the influence of LED light on plant growth appeared already two decades ago [10] and several studies on the topic were published over the years especially concerning legumes or crop plants [9,11,12,13,14], no comprehensive investigation on the behaviour of the model plant *Arabidopsis thaliana* grown under LED lights in comparison to fluorescent lights has been conducted to our knowledge.

In the present study, we aimed to compare the morphological and physiological traits of *Arabidopsis thaliana* Columbia 0 plants grown under LED lights of different intensity and spectral composition with plants cultivated under fluorescent lights in a climatic chamber. In addition, we performed gene expression analysis of all plants at day 18 after sowing (18 DAS). We observed extensive differential expression of many RNAs under different light intensities, which are partially correlated to the analysed phenotypic parameters. This in turn makes it quite clear that analysis of gene expression profiles is a good first step, but to clearly and unequivocally correlate this to phenotypic traits, in-depth biochemical investigations are mandatory.

## 2. Results

### 2.1. Plants Grown under Different Light Regimes Show Distinct Phenotypes

**Figure 1:**
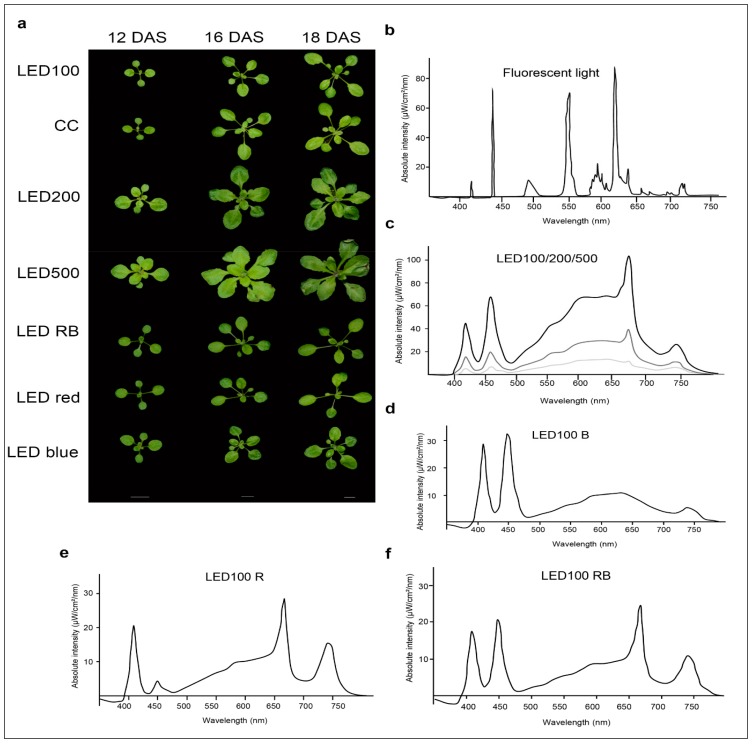
*Arabidopsis thaliana* shows distinct growth behaviour under different light and climate regimes. (**a**) Phenotypes of plants grown at either 100 µmol m^−2^ s^−1^ under white light-emitting diode (LED) light (LED100) or fluorescent light in a climatic chamber, or LED light at 200 µmol m^−2^ s^−1^ or 500 µmol m^−2^ s^−1^ LED red, LED blue or LED red/blue. Plants were grown on soil and photographed at 12, 16, and 18 days after sowing (DAS). The scale bar represents 1 cm; (**b**–**f**) Spectra of the different light regimes. Please note that the total light intensity represents the area below the spectral curves. Thus, the peak heights at the different wavelengths do not reach the value of the total output; (**b**) Spectrum of the fluorescent lamp in the climate chamber; (**c**) LED spectra at 100 (lower light grey curve), 200 (middle dark grey curve), and 500 µmol m^−2^ s^−1^ (upper black curve); (**d**) LED spectrum for LED100 with a raised portion of blue light (B); (**e**) LED spectrum for LED100 with a raised portion of red light (A); (**f**) LED spectrum for LED100 with a raised portion of red/blue light R/B).

We compared the growth behaviour of *Arabidopsis thaliana* Col 0 plants cultivated on soil under either fluorescent or LED lights at different intensities, as well as plants grown under LED light of diverse spectral composition (Figure 1b–f). 

**Figure 2:**
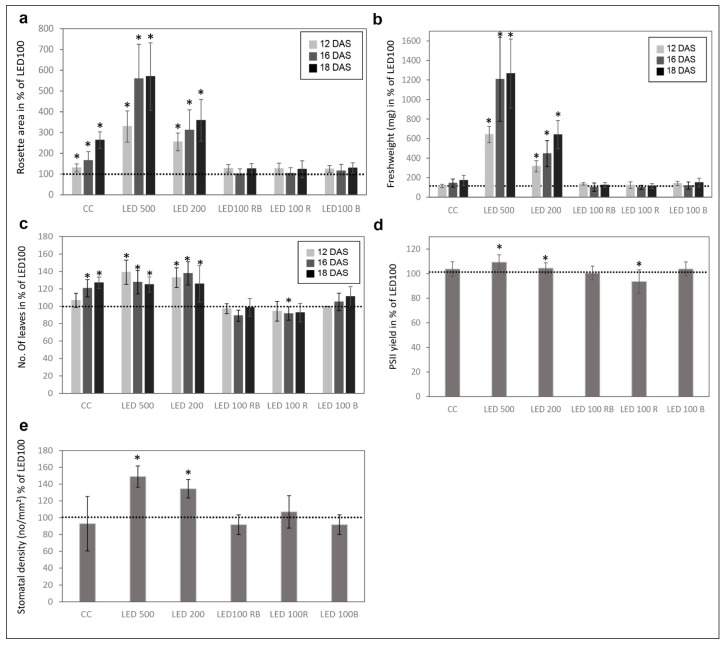
Physiological parameters of plants grown under different light regimes. (**a**) Mean rosette area in % compared to LED100 from *n* = 6 plants measured at 12, 16, and 18 DAS. The dotted line represents the value of plants grown at LED100 in all graphs; (**b**) Mean fresh weight (mg) from *n* = 6 plants at 12, 16, and 18 DAS; (**c**) Number of leaves in % compared to LED100 from *n* = 6 plants measured at 12, 16, and 18 DAS; (**d**) PSII yield from plants at 18 DAS in % compared to LED100; (**e**) Stomatal density given in number/mm^2^ from plants at 18 DAS. Significant changes compared to LED100 according to the student’s T test are indicated by an asterisk (*).

Please note that the total light intensity represents the area below the spectral curve. Thus, the peak heights at the different wavelengths do not reach the value of the total output. Pictures were taken at days 12, 16, and 18 after sowing (DAS) (Figure 1a). Our reference point in all experiments was LED100, where the wavelength distribution was adapted according to the sunlight emission spectrum as much as technically possible with our LED set-up (please refer to Figure 6b). Plants grown at the same light intensity of 100 µmol m^−2^ s^−1^ under fluorescent (Figure 1b, light grey line) or LED light in a climatic chamber (Figure 1b, dark grey line) do not show dramatically different phenotypes (Figure 1a, top and second row). Surprisingly to us, the development of plants cultivated under fluorescent lamps was faster, judged by the number of true leaves, which is significantly higher by 20–30% after 16 and 18 DAS, respectively (Figure 1a, row one and two, Figure 2c indicated by asterisks). The rosette area of plants grown in the climatic chamber differs even more at all days measured from plants cultivated in the LED chamber: 120% after 12 DAS, 160% after 16 DAS, and even 270% after 18 DAS (Figure 2a). However, the fresh weight was not significantly different from plants grown under fluorescent or LED light (Figure 2b), indicating that even though there are less leaves with a smaller area, their thickness is higher in plants grown under LED lamps, which could be due to the comparatively higher amount of blue light.

Raising the light intensity in the LED chamber to 200 µmol or 500 µmol, respectively, under constant spectral distribution led as expected to faster development (more leaves, bolting visible) and growth (bigger leaf areas and higher fresh weight) (Figure 1a, third and fourth row, Figure 2a–c). Plants subjected to high light intensities accumulated much more fresh weight: in the case of LED200 about 600%, at LED500 even up to 1300% compared to LED100. To exclude any adverse effects of heat emitted by the LED lamps under high light intensities, we measured the temperature at the leaf level and found it to be at 23 °C, thus we can rule out that our observations on phenotypic or gene expression levels are caused by elevated temperatures.

In parallel, we analysed the effects of spectral quality while keeping the total light intensity at a constant 100 µmol. Therefore, white light (3 K) was lowered in all three conditions. To achieve a light quality with a relatively high portion of red light (for simplicity we call it “red, R”), the electrical output of the 660 nm LEDs was enhanced and the 730 nm LEDs were kept unchanged, while the UV (395 nm) and blue light (440 nm) LEDs were lowered in their output. To bring about a high portion of blue light (named “blue, B”), UV and blue LED output was raised, while red and far red light LEDs were lowered in their output. In a combination of both settings (we call it “red blue, RB”), we achieved spectral peaks at 440 nm as well as 660/730 nm. An overview about the spectral composition, measured as fluence rates, is depicted in Figure 1d–f. By this we ensured that the total light intensity (meaning the fluence rate) was kept at 100 µmol in all conditions. Plants grown under a combination of red and blue (RB) LED light compared to LED100 were growing very similarly (Figure 1a, top and fifth panel, Figure 2). Red light seemingly led to marginally slower growth, however this was not represented by significant differences in the measured phenotypical characteristics (Figure 1a and Figure 2). Blue light resulted in a more compact-appearing phenotype and shorter petioles, whereas neither rosette area nor fresh weight nor number of leaves were different from LED100. Thus, we could clearly demonstrate that by using LED lamps with distinct spectral characteristics we can influence the growth behaviour concerning the appearance of Arabidopsis plants. 

### 2.2. Physiological Traits Differ in Plants Grown under Different Light Regimes

In addition to visual analyses, we investigated several physiological traits of the differently cultivated plants at 18 DAS (Figure 2d,e). The photosynthetic performance showed small but clear differences between the plant populations (Figure 2d). The PSII yield was higher in plants from LED200 and 500 and red light. Thus, the higher number of leaves and greater leaf area in the plants from the climate chamber with fluorescent lights does not result in a higher photosynthesis rate compared to plants from the same light intensity under LED lamps. Consequently, these leaves seem to feature either a lower number of photosystems per area or less active ones. In contrast, high light intensity leads to overall bigger plants and better photosynthetic performance. 

Since stomata development is known to be light dependent [15], we analysed the stomatal density in plants from all conditions at 18 DAS (Figure 2e). LED200 and LED500 plants had an evidently higher stoma density (140 and 130% of LED100, respectively). All other plants were similar to the ones from LED100 concerning the number of stomata, indicating that for this physiological trait light fluence rate is more important than wavelength composition. This observation is in line with previously reported data that high irradiation increases the stomatal density [16]. It was found in the same study that monochromatic red light also influences this trait, but probably since we did not apply monochromatic light, but rather increased the portion of red light with still white and blue light present, we did not detect a similar effect.

### 2.3. Affymetrix Analysis Reveals Differentially Regulated RNA Expression

**Figure 3:**
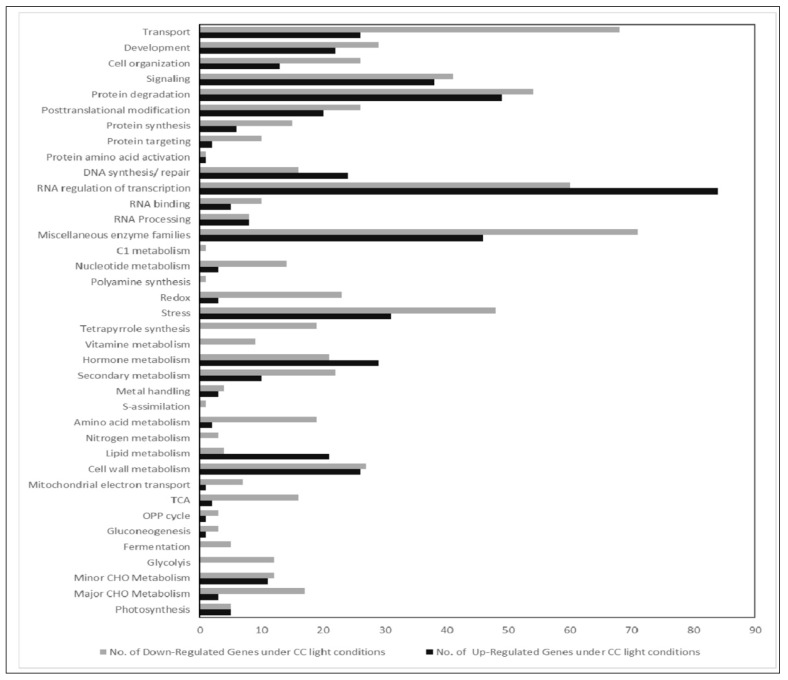
Comparative gene expression analysis of RNA isolated from plants grown in LED100, climate chamber. Bar chart of up (black bars) and down (grey bars) regulated genes from plants grown in the climate chamber.

**Figure 4:**
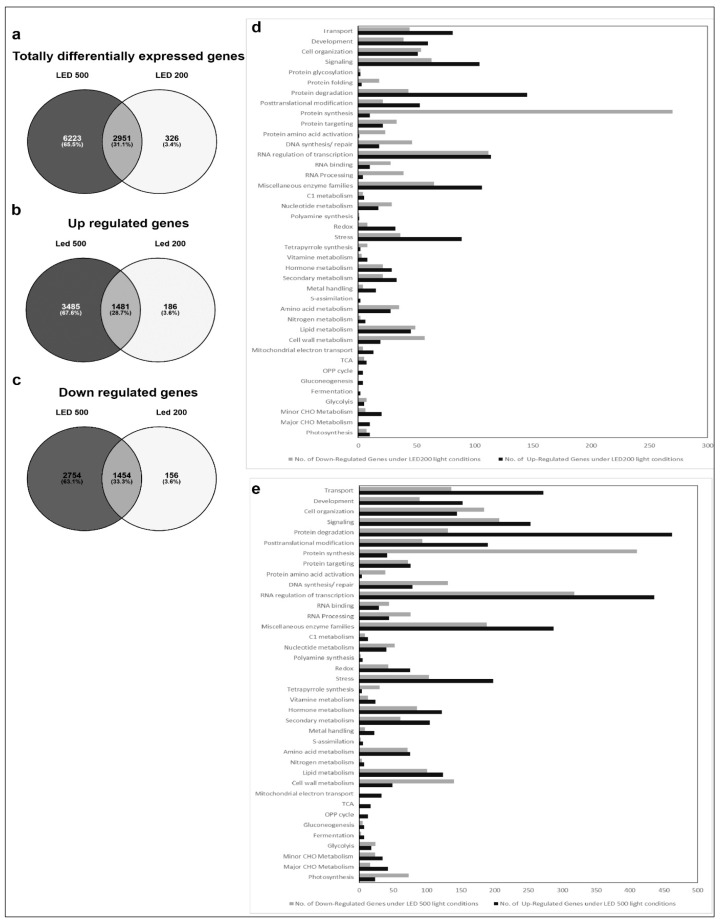
Comparative gene expression analysis of RNA isolated from plants grown in LED100, LED200 and LED500. (**a**–**c**) Venn diagrams of a total differentially expressed; (**b**) up regulated; (**c**) down regulated genes; (**d**) Bar chart of up (black bars) and down (grey bars) regulated genes from plants grown in LED200; (**e**) Bar chart of up (black bars) and down (grey bars) regulated genes from plants grown in LED500.

**Figure 5:**
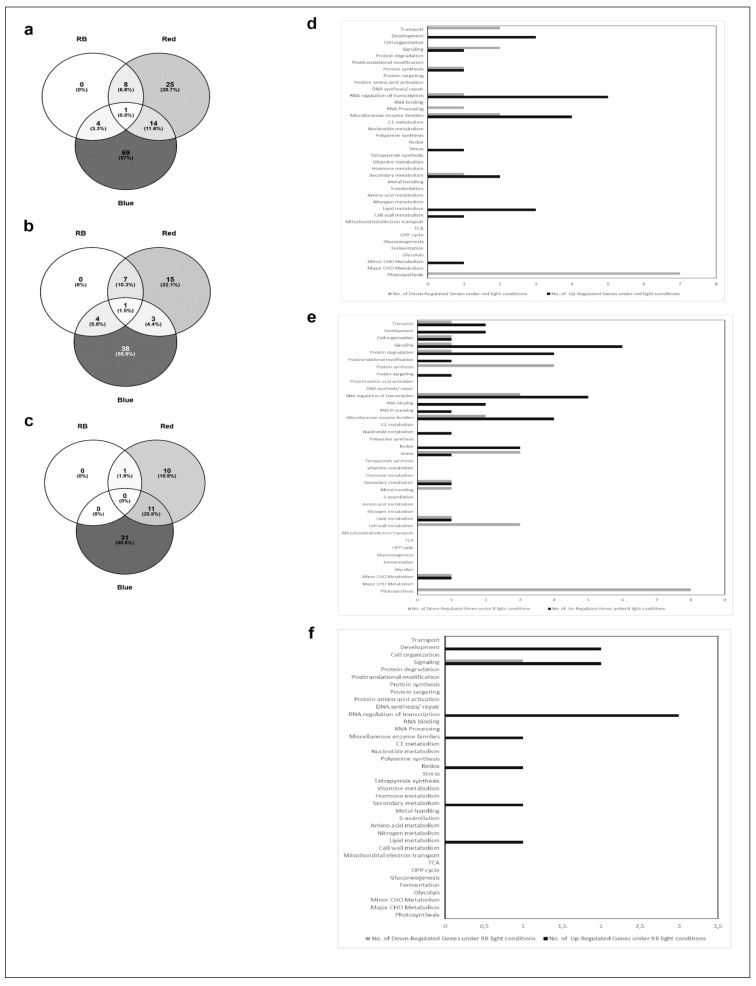
Comparative gene expression analysis of RNA isolated from plants grown in LED100, LED R, LED B, LED RB. (**a**–**c**) Venn diagrams of a total differentially expressed; (**b**) up regulated; (**c**) down regulated genes; (**d**) Bar chart of up (black bars) and down (grey bars) regulated genes from plants grown LED R; (**e**) Bar chart of up (black bars) and down (grey bars) regulated genes from plants grown in LED B; (**f**) Bar chart of up (black bars) and down (grey bars) regulated genes from plants grown in LED RB.

In an attempt to correlate the monitored morphological and physiological features with gene expression, we performed Affymetrix analysis with RNA isolated from leaves harvested at 18 DAS. Genes were defined as differentially regulated the if the p-value was lower than 0.05. As for the data in Figure 2, LED100 was applied as the reference point in all evaluations. We then compiled separate groups for comparative analyses: 1. climate chamber with fluorescent lamps and LED lights; 2. LED200 and LED500 as well as 3. R, B, and RB (Figure 3, Figure 4 and Figure 5). For each group except the first, we generated Venn diagrams of total, up- and down-regulated genes (Figure 4a–c and Figure 5a–c), respectively. We executed MapMan analyses for all groups in which the bins are represented as bar charts (Figure 3, Figure 4d,e and Figure 5d,e).

For the first group (climate chamber with fluorescent lights compared to LED100), we found about 200 genes differentially expressed with a fold change (FC) >2 (Supplemental Table S1). We can, however, not completely exclude that some effects on gene expression between plants in the climate chamber with fluorescent lamps and plants in the chamber with LED lights are due to the different climate chambers. These are of extremely similar build, volume, and set-up, but not of identical make.

Noticeably, the highest number of genes regulated is to be found in the MapMan bin RNA regulation and transcription, indicating that gene expression in general is clearly influenced by the different light quality. Apart from that, many genes in the groups of transport, protein homeostasis, and enzymes are differentially expressed, though no single pathway stands out. Since the appearance of the plants is so similar, this indicates that diverse expression of genes is not necessarily reflected in a distinctive visible phenotype. Nevertheless, one needs to be aware that applying different light sources leads to fundamental changes in the transcriptome, even though the overall characteristics of plants grown under disparate types of lamps are not easily distinguishable.

In the second group, comprising plants from the different light intensities LED200 and LED500 (Figure 4), a total of 9500 RNAs is differentially expressed compared to LED100, demonstrating that many physiological activities are altered under higher light intensities, which is clearly reflected by the pronounced growth of the plants. From these, 1454 genes are down regulated in LED200 (Figure 4c) as well as in LED500 (Figure 4b) and 1481 are up regulated in both categories. In inference, this means that 2754 RNAs are less-expressed solely in plants from LED500, and 156 RNAs from LED200. Down-regulated exclusively in LED500 were 3485 genes, restricted to LED200 were 186 genes (Figure 4a–c). Thus, higher light intensity leads in general to a greater response in RNA level. The fact that about 30% of all genes are regulated under both conditions with the FC bigger at higher intensities demonstrates the reproducibility of our approach. Concerning the distribution of these genes across the MapMan bins, the picture is somewhat different from the first group (Figure 4d,e). Focussing on genes with a FC > 2 (Supplementary Table S1) reveals that most differentially expressed RNAs belong to the bin proteins. The largest portion of up regulated RNAs from this bin code for proteins involved in the processes of degradation, followed by modification. This implicates that under higher light intensities protein turnover might be faster. Surprisingly, among the lower expressed RNAs, most can be assigned to the class of synthesis of ribosomal proteins, which suggests that while plants grown under higher light intensities perform protein degradation to a larger extent, they do not counteract that by increasing the translation machinery to produce more proteins de novo. Since these plants clearly develop faster and have significantly more biomass, the phenotypic observations seem quite contradictory to the RNA expression patterns. However, it may well be that the ribosomal proteins are protected against degradation and are simply more stable than many other proteins. Thus, this RNA expression pattern does not necessarily indicate a lower protein synthesis activity per se. The highest fold change can be observed for BAM5 under LED500 which codes for a putative ß-amylase (FC = 157), while other genes from the starch degradation pathway are less drastically but still prominently up-regulated (FC = 4–13). Considering the accelerated growth under the applied conditions, this nicely reflects the elevated need for carbohydrates. In the same line, we can interpret the increased expression of genes related to transport functions, since many metabolites need to be transported within the plant to ensure growth. Since plant growth, among many other complex processes includes cellular expansion, the up-regulation of auxin-related genes fits into this picture [17]. High light intensities of especially 500 µmol also lead to increased generation of reactive oxygen species, consequently we find RNAs belonging to the bins stress and redox at a higher expression level in plants from LED200 and LED500. Since the plants have a generally healthy appearance, this up-regulation does not necessarily indicate stress, but acclimation mechanisms to prevent stress-related damage.

In the last group, we compared gene expression in plants grown under red light, blue light, or a combination of red and blue, all supplemented with white light at 25% of the default intensity LED100, thereby ensuring a total intensity of 100 µmol m^−2^ s^−1^. In contrast to our expectations, very few genes were differentially expressed compared to LED100 (Figure 5). It has been known for some time that red light induces the phytochrome system, whereas blue light results in the induction of phototropins and cryptochromes (for review, see [2]). However, all these previous studies were conducted after implementing pulses of the respective wavelengths, immediately followed by gene expression analysis, while we grew the plants continuously under the indicated spectral regimes for 18 days. This obviously leads to long-term acclimation and thereby results in a different pattern of gene expression, when the peak of light-induced expression might already have flattened. A total of only 121 RNAs was regulated in expression in relation to LED100 (Figure 5a). A single gene is commonly up-regulated in all conditions which belongs to the signalling bin. Nothing more is known about its function, though it is annotated as a receptor kinase (Supplementary table). This gene was found to be significantly regulated in many conditions, thus it’s questionable if this represents a specific effect. 38 RNAs appear at a higher level solely in blue light, most of them distributed among the bins signalling, transcription, protein degradation, enzymes, and redox (Figure 5e). Expressed at a lower level, we found RNAs mainly concerning photosynthesis-related functions, which is surprising in view of our PSII yield measurements that show a slightly higher, though not significantly, activity of photosystem II (Figure 2d). Since the effected genes are coding for PSI, PSII, and NDH complex components, the RNA expression level obviously does not correspond to process performance in this case. Support for this conclusion comes from studies of translational activity compared to gene expression, which is not necessarily coupled [18]. Other down-regulated genes primarily participate in protein synthesis, transcription, stress, and cell wall metabolism (Figure 5e). 

Red light leads to the elevated expression of genes from development, signalling, protein synthesis, transcription, miscellaneous enzymes, stress/acclimation, secondary metabolism, lipid metabolism, and cell wall and CHO metabolism (Figure 5d). Less transcribed under red light are predominantly genes belonging to the category of photosynthesis, concerning specifically PSII constituents (Supplementary table), which in this case is in line with the pulse-amplitude modulations (PAM) measurements (Figure 2d). The remaining down-regulated genes are involved in transport, signalling, protein synthesis, transcription and RNA processing, enzymes, and secondary and CHO metabolism. 

Combining red and blue light results in a revocation of effects from red or blue light on gene expression level. While the plants phenotypically more resemble the ones cultivated under only red light, differential RNA synthesis overlaps with red and blue light treatment (Figure 5f). Interestingly, the number of genes that are regulated here is lower than under red or blue light, respectively (Figure 5a). The respective genes appear consistently as differentially expressed throughout all conditions analysed, indicating that these might represent key genes regulated upon specific treatments with red and blue light.

## 3. Discussion

Altogether, we can conclude that growth behaviour of *Arabidopsis thaliana* Col 0 is clearly influenced by light quantity and quality (Figure 1). This in itself is not a novel concept, but our conditions for plant growth were quite unusual, in that we did not treat the plants with short periods of different wave lengths and look for short-term effects, but kept them under high portions of red, blue, or red/blue light throughout the whole experiment.

Provided with sufficient nutrients to build cellular material, high light intensities led to about ten-times faster growth and development (LED500 compared to LED100). In spite of the high radiation, plants cultivated at 500 µmol appeared healthy and were obviously not stressed, which would have been reflected in anthocyanin accumulation and thus a reddish colour [19]. One should keep that in mind when analysing the effect of high light on plant physiology, so that general stress reactions are not confused with high light specific acclimatory processes. 

Exposing the plants to higher doses of blue light also had visible effects on their phenotypes: it led to more stunted growth with short petioles but more leaves. In a combination of red and blue light, the effect of red light prevails and plants looked very similar to those raised under red light only. It was shown in several physiological studies that for the fine-tuning of light responses, the photoreceptors for red and blue light act synergistically [20,21]; under the conditions applied in our study, signalling from red light receptors seems to have a dominant effect.

Though LED light still has profound consequences for gene expression, phenotypically the plants are not dramatically different. This observation is in line with a previous publication where several laboratories strived to achieve comparability in their plant growth setups and found that, especially on the gene expression level, the variation was already quite high between individual plants from the same growth cabinet which seemed to originate from changes in the microenvironment [22]. Gene expression obviously reacts much faster than phenotypic appearance, which one needs to take into consideration upon interpreting gene expression data in correlation to phenotype or biochemical activity.

Plants incubated under 100 µmol m^−2^ s^−1^ LED light displayed slightly slower growth compared to plants from the climate chamber equipped with fluorescent lights (Figure 1a). Considering that the LED light features a nearly optimal wavelength composition with regard to comparability to the natural sun light and in contrast to the spectral distribution provided by fluorescent lamps (Figure 1b–d), we would have anticipated accelerated development under LED light. However, on closer scrutiny of the spectra and super-positioning of both emission curves (Figure 1b,c), it becomes clear that the very narrow but high peaks emitted by the fluorescent lamps in the blue and red light range cover a very similar area as the broader but lower peaks from the LED lights. This probably results in roughly the same or even higher amount of photosynthetically active radiation emitted by the fluorescent lamps at 100 µmol m^−2^ s^−1^ compared to our setup of the LEDs. Thus, in a next step we will further vary the spectral distribution of the LED lights at 100 µmol m^−2^ s^−1^ and compare those to fluorescent lamps. 

In this study, we could clearly show that manipulation of the wavelengths and/or intensity of the applied light source can influence the growth behaviour and gene expression of Arabidopsis plants. By using LEDs, it is possible to roughly mimic the natural light conditions and study plant growth of different genotypes as closely as possible to what would happen in the field. This is a considerable advantage of LEDs over other commercial light sources, which feature fixed emission spectra. Furthermore, fluorescent lamps exhibit changes in emission quality after a relatively short life time, whereas LEDs provide stable light quality and quantity over extended time periods. In addition, using LEDs avoids the added stress factor of excessive heat produced by commercial lamps, which drastically influences gene expression and development. This is currently a common problem when studying plant response to high light treatment, because it leads unavoidably to a higher leaf temperature. In our setup, the temperature at leaf level was constantly 23 °C under 500 µmol LED light. Furthermore, the energy consumption of LEDs is much lower than that of any other available light source, thus substantially reducing the running costs of climate chambers/greenhouses.

## 4. Materials and Methods

### 4.1. Plant Materials and Growth Conditions

All experiments were performed on *Arabidopsis thaliana* ecotype Columbia 0. Seeds of Arabidopsis were sown on soil and vernalized for two days at 4 °C. Then, the plants were transferred to environmentally controlled growth chambers under different light treatments: climatic chamber (CC) refers to a growth chamber equipped with fluorescent lights and LED refers to a growth chamber supplied with light emitting diodes. In addition, the spectral output of the fluorescent lamps was determined for a new lamp compared to one of about six months of age (Supplemental Figure S1) to provide an idea of when to change lamps for comparable experiments.

The LED lights had the following specifications: OSRAM Osslon SSL Far red 730, OSRAM Osslon SSL Deep blue 450, OSRAM Osslon SSL hyper red 660, OSRAM Osslon SSL light colour 3.000 K (Osram, Munich, Germany), Edison Federal 3535-UVA 395–410 (LED-Tech, Moers, Germany). Environmental conditions in the chambers were set at a 22 °C/18 °C day/night temperature, air relative humidity of 50–65%, and a 16 h photoperiod. In total 50 seedlings per condition and light treatment were used. Plants were watered by subirrigation and fertilised upon every second watering, usually every 2 to 3 days, depending on the growth stage. The spectral distributions for the seven light treatments are shown in Figure 1b–f and were determined using a calibrated OceanOptics (Duiven, Netherlands ) spectrometer at the day of sowing. In short, we programmed the desired light intensities into the software of the LED chamber. The actual output is controlled by a built-in sensor within the chamber. The spectra were recorded at the setting for absolute intensities (µW/cm^2^/nm), which is the most precise method. Therefore, we could monitor the total light output at the same time as the distribution over the wavelength spectrum.

### 4.2. Phenotypic Analysis

To closely monitor the growth of Arabidopsis plants under different light conditions, a detailed phenotypic analysis was conducted. For visual monitoring, eight plants per condition were photographed in two-day intervals. Depicted in Figure 1 are exemplary plants from 12 DAS, 16 DAS, and 18 DAS.

### 4.3. Spectroscopic Analysis

Chlorophyll a fluorescence of Arabidopsis leaves was measured using a pulse-modulated fluorimeter (Imaging PAM Mini; Walz) and the PSII yield was determined according to the manufacturer’s instructions.

### 4.4. Stomata Density

Three fully-expanded rosette leaves from 3 individual plants were collected into 70% ethanol, cleared from chlorophyll overnight at room temperature, and stored at 4 °C as needed and then further cleared in chloral hydrate solution (chloral hydrate:water:glycerol (8:2:1, *w*/*v*/*w*)). Differential Interference Microscopy (DIC) images of the abaxial surface were captured with a Leica DM1000 microscope (Leica Microystems, Wetzlar, Germany)at 40× magnification. Stomatal density (mm^2^) was manually counted for all pictures and all leaves.

### 4.5. Fresh Weight Determination

Total fresh weight data were obtained by carefully removing all of the leaves including petioles from six Arabidopsis plants from each light condition. All of the individual leaves were immediately weighed to obtain fresh weight data. Data were subjected to statistical analyses (Student’s *t* Test). 

### 4.6. Rosette Area Determination

Rosette areas from eight plants per condition were determined graphically with ImageJ and the data were statistically evaluated by Student’s *t* Test.

### 4.7. Transcriptomic Profiling Using Affymetrix ATH1 Microarray

For microarray analysis, leaves of 18-day-old Arabidopsis plants were used. To provide biological replicates, three samples were harvested from 10 individual plants. Total RNA was extracted using the Plant RNeasy Extraction kit (Qiagen, Hilden Germany). RNA concentration, purity, and integrity were determined. The purified RNA (200 ng) was used to produce biotinylated cRNA probes by using an Affymetrix 3′-IVT Express kit (Affymetrix, High Wycombe, UK) according to the manufacturer’s instructions. A total of 12 µg biotinylated cRNA was fragmented and hybridized to GeneChip *Arabidopsis* ATH1 arrays containing 22,810 probe sets. Washing and staining were done on an Affymetrix GeneChip Fluidics Station 250. The array chips were scanned using an Affymetrix GeneArray Scanner 3000. Raw signal intensity values (CEL files) were computed from the scanned array images using the Affymetrix GeneChip Command Console 3.0 (Affymetrix, Santa Clara, USA). For quality checking and normalisation, the raw intensity values were processed with Robin software [23] using default settings. Specifically, for background correction, the robust multiarray average normalization method [24] was performed across all arrays. Statistical analysis of differential gene expression was carried out using the linear model-based approach developed by Smyth [25]. The obtained *p* values were corrected for multiple testing using the strategy described by Benjamini and Hochberg [26] separately for each of the comparisons made. Genes that showed a *p*-value lower than 0.05 were considered significantly differentially expressed. The normalised log2 values where then used to compare the transcriptomic changes using MapMan. Based on MapMan BINs [27] the significantly expressed genes were functionally annotated. In total, we analysed the following comparisons: LED100 versus Climate chamber, LED100 versus LED500, LED100 versus LED200, LED100 versus LED RB, LED100 versus LED R, and LED100 versus LED B.

## 5. Conclusions

**Figure 6:**
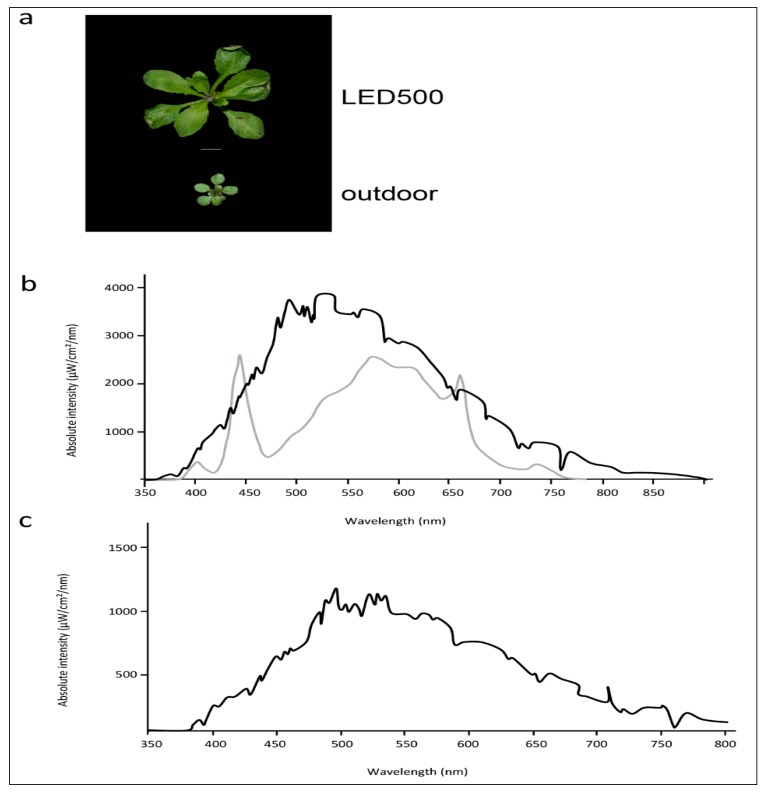
Arabidopsis grows better under completely controlled environmental conditions. (**a**) Phenotypes of plants grown under LED500 or outdoor. Plants were grown on soil and photographed at 18 DAS or, in case of the plant grown outdoor, 38 DAS, respectively. The scale bar represents 1 cm. Please note that the picture of LED500 is identical to Figure 1; (**b**) Emission spectra of LED500 (lower grey line) and natural light at sunny conditions (upper black line); (**c**) Emission spectrum of natural light at cloudy conditions.

While plant growth under fluorescent lights leads to acceptable results and healthy plants, the spectral distribution provided by those lamps is far from comparable to natural sun light. If we strive to observe and analyse our plants as close as possible to the conditions outside in their natural habitat, we need to apply variable light sources such as LED lights where single wavelengths can be adjusted and freely combined. As a further subject for thought, in addition to our study conducted with plants cultivated in climatic chambers, we grew Arabidopsis outside under completely natural conditions (Figure 6a). These plants are obviously smaller and more compact than the plants from climatic chambers, while the leaf phenotypes concerning petiole length and leaf area are quite similar to the plants grown at LED500. The spectral distribution of LED500 was adjusted as close as technically possible to full sun light (Figure 6b). Of course, one needs to keep in mind that the plants grown in the field were subjected to constantly changing humidity and temperature as well as very variable light conditions due to weather changes, which we didn’t monitor continuously. As depicted in Figure 6b,c, the intensity alone changed immensely from cloudy conditions to full sun light. Thus, the divergent growth behaviour is not solely due to different spectral quality. With the use of LED lights, we can move forward towards our goal of observing plant growth and behaviour under conditions mimicking those occurring in nature—especially when further technical advance will allow us to synchronise light conditions from outside with those in climatic chambers.

## Supplementary Materials

The following are available online at www.mdpi.com/2223-7747/6/2/24/s1, Figure S1: Comparison of the spectral output of a new fluorescent lamp (blue line) with one approximately six months old (red line), Table S1: Gene Expression.

Supplementary File 1

Supplementary File 2

## Abbreviations

LED	light emitting diode
DAS	days after sowing
R	high portion of red light
B	high portion of blue light
RB	high portions of red and blue light
