# Supplementary material for: Comparative Phenotypical and Molecular Analyses of Arabidopsis Grown under Fluorescent and LED Light
Source: Plants (Basel). 2017 Jun 13;6(2):24. doi: 10.3390/plants6020024 (PMC5489796; doi:10.3390/plants6020024)
Supplement: Supplementary File 1 [file plants-06-00024-s001.pdf]

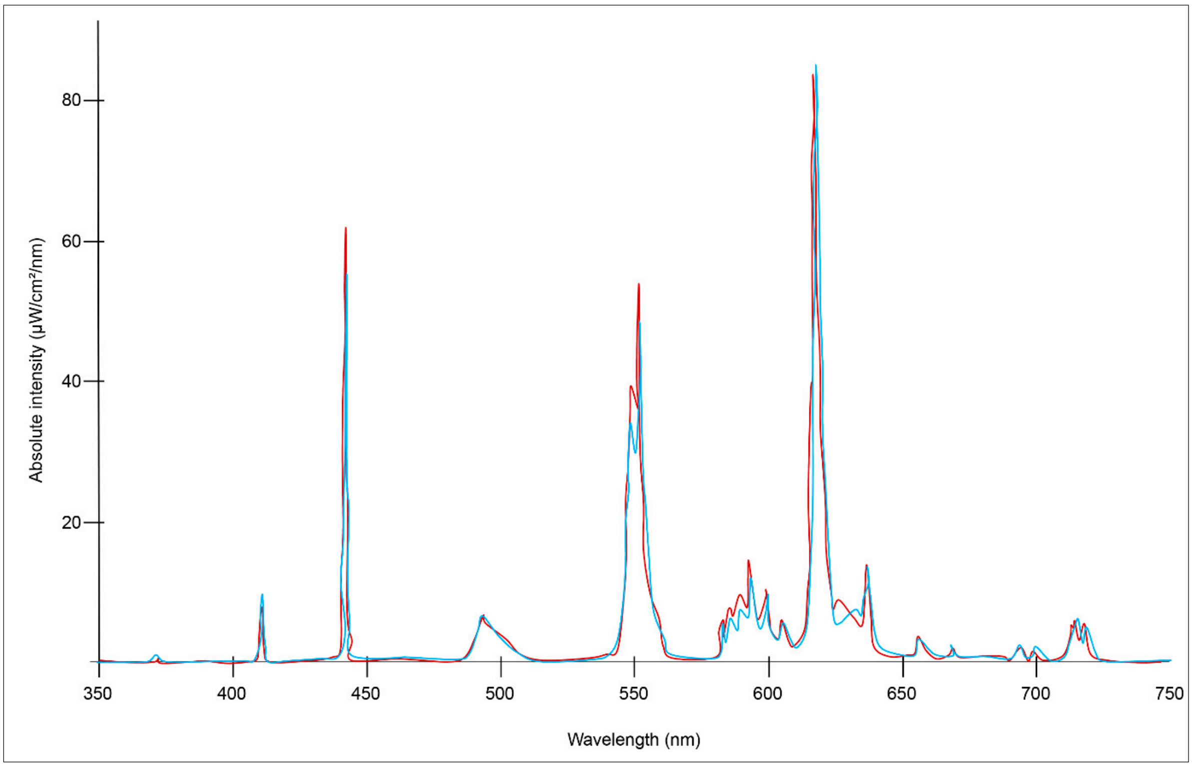

Figure S1: Comparison of the spectral output of a new fluorescent lamp (blue line) with one approximately six months old (red line).
